# Strategies and barriers to implementing physically active teaching in universities from the perspective of lecturers: a qualitative study

**DOI:** 10.1186/s12889-025-22075-x

**Published:** 2025-03-04

**Authors:** Robert Rupp, Birgit Wallmann-Sperlich, Jens Bucksch

**Affiliations:** 1 Department of Prevention and Health Promotion, Heidelberg University of Education, Keplerstraße 87, Heidelberg, 69120 Germany; 2 Institute of Sport Science, University of Würzburg, Judenbühlweg 11, Würzburg, 97082 Germany

**Keywords:** Stealth intervention, Sedentary behavior, University students, Sitting, University teaching, Teaching methods, Active learning, Pedagogy, Higher education, Physically active learning (PAL)

## Abstract

**Background:**

University students accumulate lots of sedentary time without interrupting and comprehensive approaches to reduce time spent sedentary are lacking. The implementation of physically active university teaching needs practicable approaches and the support by lecturers. However, there is little research on which physically activating strategies lecturers actually use and what barriers they become aware to implement these. This exploratory, qualitative study aims to identify physically activating strategies as well as barriers for reducing sitting time and physically active university teaching from the perspective of lecturers.

**Methods:**

We conducted semi-structured interviews with 16 lecturers to explore potential physically activating strategies in university teaching, assess their degree of utilization, and identify barriers to implementation. The data were analysed by a structured content analysis of the interview transcripts using MAXQDA 2020 software.

**Results:**

Physically activating strategies are hardly known among university lecturers and are seldomly used on a regular and conscious basis. We identified two types of strategies with physically activating measures and teaching methods. Lecturers highlighted two specific types of physically activating measures: physical activity breaks and the use of physically activating furniture. All together, we identified 18 distinct teaching methods (e.g. group work, gallery walk) that integrate learning processes with physical activity in a pedagogical-didactic manner. The main barriers to implementation identified were lack of space, lack of time, students’ unwillingness to move; organizational social norms, and lecturers’ uncertainty about how to implement these strategies effectively.

**Conclusions:**

University lecturers are generally unfamiliar with and rarely use physically activating strategies to reduce sedentary behavior in students. However, lecturers identified 18 potential teaching methods that integrate physical activity with pedagogical-didactic principles, offering a new approach to physically active university teaching. These methods present an untapped potential for the low-threshold integration of physical activity and breaks from sitting into university teaching, aligning with "stealth health" strategies that incidentally promote health while assuring a “high-quality education” as the core concern of higher education teaching. Understanding and addressing the barriers to implementation, such as lack of space, time, social and organizational norms, is crucial for the effective planning and implementation of interventions.

## Background

University teaching and learning largely occur while being sedentary, usually without interruptions [1–4]. According to a systematic review, university students spend an average of 11 h a day sedentary [5]. However, sedentary behavior within university settings has received limited attentiveness. This might be traced back to the normative belief, that teaching–learning arrangements while sedentary is the appropriate and culturally ingrained method for absorbing, accessing, or reflecting on information [6]. Even more learning is often accompanied by a neglect of physicality [7]. This well-established "anti-body attitude" maintained by universities alongside the resulting dichotomy between body and mind in the learning process may be identified as a fundamental malaise of Western education [8].

From a health perspective, long episodes of being sedentary without interruptions increase the risk of chronic diseases [9–13]. Further on, regularly interrupting sedentary behavior while learning and in teaching arrangement also seems to have a positive impact to cognitive functioning and academic performance. A study concludes, that college students’ cognitive functions related to academic performance improve when prolonged sitting is interrupted every 20 min [14]. At the same time, prolonged periods of uninterrupted sitting during university courses are associated with reduced attention and focus among students [15]. Further studies have demonstrated that the introduction and utilization of standing desks in seminar rooms led to increased attention and decreased restlessness among university students. Moreover, some students reported more focused participation, increased engagement and reduced feeling of tiredness and boredom during seminars [1, 4]. In addition, the introduction and implementation of physical activity breaks or simple standing breaks in university classes have already been shown to enhance students’ self-perceived physical, mental, and cognitive states [3, 16–18]. Studies from the school and working context underpin that physical activation during school lessons and during working time support cognitive functioning and performance [19–23].

Despite these positive findings sedentary behavior in university teaching seems to be highly prevalent and solutions to promote active learning-teaching arrangements have to be taken more seriously into account. At a descriptive level, two main approaches have been observed to integrate more sitting interruptions and physical activity bouts into university teaching: the use of physical activity/standing breaks and access to physically activating furniture such as sit-stand desks [17]. In the following, we use the term *physically activating measures* to summarise these two conventional approaches. However, interrupting sitting via physical activity/standing breaks presents disadvantages from a pedagogical-didactic perspective. These breaks are not aligned with the subject matter of a teaching course in terms of content or timing, potentially reducing learning time and disrupting the teaching–learning flow [18, 24, 25]. Consequently, the primary task of (high) school/university teaching (effective learning) is postponed in favour of promoting physical activity [24, 26]. The use of physically activating furniture represents alternative answers towards promoting physically active university teaching. Compared to physical activity breaks or standing breaks, sit-stand desks offer a more favourable balance in terms of learning time. They present opportunities for integrating physical activity into university teaching while preserving learning time by synchronizing physical activity in class with learning [26]. However, their adoption entails cost implications [27].

**Fig. 1:**
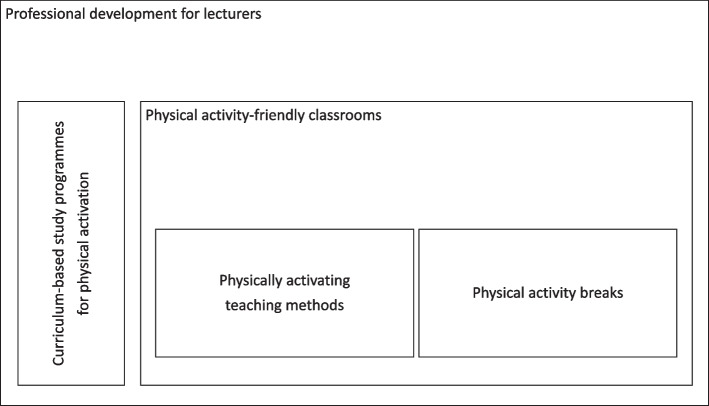
Heidelberg model of physically active teaching — modified according to [6], reproduced with permission from SNCSC

In terms of the pedagogical-didactic disadvantages and an acceptance of more physical activation and physical activity during learning-teaching arrangements from the perspective of lecturers the search for further approaches is necessary. The Heidelberg model of physically active teaching draws on the approaches and preliminary considerations presented above and highlights new directions [6]. On the theoretical basis of the social-ecological approach, the Heidelberg model presents a multi-perspective and multimodal conceptual framework that guides our study. Regarding considerations of resource-oriented pedagogy and the theory of psychological needs, the model focuses on the human design of teaching and learning processes [28]. Five practical modules are proposed, which are illustrated in Fig. 1: 1) physical activity-friendly classrooms, 2) physically activating teaching methods, 3) physical activity breaks, 4) Curriculum-based study programmes for physical activation, 5) professional development for lecturers. While the first module refers to the spatial design of seminar rooms, modules 2 and 3 deal with concrete implementation tips for lecturers to enable them to design seminars that encourage physical activity. In addition, essential modules for professionalisation are integrated to qualify lecturers and students. The physically activating teaching methods suggested in the Heidelberg model are of key relevance to our study. These methods promote interruptions to sedentary behaviour and increase everyday physical activity while preserving learning time. They can be integrated into university teaching in a way that is primarily congruent with the objective and close to the subject matter. For example, when students stand up and walk around the classroom as part of a "Gallery Walk" to view and discuss the exhibited work results of other students, the resulting physical activity does not represent an interruption of the teaching–learning process, but the physically active time is also learning time and pursues primary pedagogical-didactic goals of student activation. Physical activity is therefore not used to interrupt teaching–learning processes to reduce learning time but becomes a teaching principle that accompanies and supports learning [6, 24].

To the best of our knowledge there is a lack of studies examining the perspectives of university lecturers to interrupt sedentary behavior in terms of their ideas and barriers to implement physically activating teaching methods as well as using other measures like breaks or activating furniture. We know from the school context that the success of integrating physical activity as an additional topic in the teaching program is of limited effectiveness [29]. One German study also showed that school teachers have a critical attitude and reluctance towards physically activating measures if the primary focus is on health promotion [26]. More promising are concepts which focus on how physical activity and physical activation align the primary aim of a high quality pedagogical-didactical performance [30]. This corresponds to the basic idea of stealth health interventions, in which physical activity is a side effect and co-benefit of the intervention, which is primarily focused on context-specific core objectives [24, 31]. The approach of physically activating teaching methods outlined above can be seen as an ideal example of this, as they activate university students both cognitively and physically for the primary goal of optimising learning processes [6, 24].

Therefore, the aim of our study was to investigate lecturers’ knowledge and perception of physically activating teaching methods as well as other measures like physical activity breaks or activating furniture to implement more physical activity into learning-teaching arrangements in general. We were also interested in whether the physically activating teaching methods and measures were regularly implemented in teaching practice. In addition, the view of lecturers on the barriers to implementation is central here, as their pedagogical-didactic preparation also determines whether physically activating teaching methods, sit-stand desks or physical activity breaks are used in seminars and lectures.

## Methods

This exploratory study used a qualitative methodological approach to explore new ideas from the subjective views of lecturers as they play a key role for implementing a new routine in teaching–learning arrangements.

### Setting

The qualitative interview study was conducted at the Heidelberg University of Education which is designed to train and qualify prospective teachers for a wide range of teaching careers in elementary, secondary, and special needs education. The University of Education also offers training to other types of educators, including cooperative bachelor’s and master’s degree programs in pedagogy for engineers to attain a qualification for teaching in vocational schools. Further programs include Early Childhood Education (B.A.), Health Promotion (B.A.; M.A.), Educational Sciences (M.A.), E-Learning and Media Education (M.A.) as well as Engineering Education (M.Sc.). Approximately 300 permanent lecturers teach more than 4,700 students at the Heidelberg University of Education.

At the time of the interview survey, no program for reducing sedentary time or physically activation in teaching had yet been implemented at this university. Only in the Department of Physical Education were individual lecturers pre-sensitized to the topic due to a physically-activating school teaching project. This department also had a movement-friendly seminar room at the university that was partially furnished with sit-stand desks.

### Sample and recruitment

The interviewees were lecturers at the Heidelberg University of Education. They were recruited by the first author (RR) via direct contact and e-mail inquiries in a purposive sampling process. This means lecturers were selected in such a way that different levels (professors, academic staff) and subject areas (Educational Sciences, Mathematics, Physics, Special Education, Early Childhood and Elementary Education, German, History, Everyday Culture and Health, Physical Education and Sociology) were covered. Recruitment was iterative and ended when data saturation was reached (*n* = 16), i.e. the collection of new qualitative data did not produce any new codes. Up to this point, 26 lecturers had been requested for an interview and 10 lecturers refused to participate mostly due to lack of time. Interested lecturers were informed verbally and in a written form about the aims of the qualitative study, the data protection policy, and the interview conditions to obtain their informed consent. The study complies with ethical and legal data protection regulations and was approved by the Ethics Committee of the Institute of Sport Science of the University of Würzburg (EV2024/2–2305).

#### Development of the interview guide

The interview guide was developed according to the SPSS (German acronym for collecting, checking, sorting, and prioritizing) method of Helfferich [32], and based on the research team's experience with qualitative research. SPSS method is an established procedure for developing and ensuring the validity of interview guides. The procedure consists of four steps: collecting, checking, sorting and subsuming. In the first step, we collected terms in our working group that was concerned with the subject of the research. Inspired by similar qualitative analyses from earlier studies in the school context [33, 34] and from the workplace context [35], we provided further ideas for possible terms and questions in the interview guide. The resulting collection of terms and questions was then critically discussed in the working group and, after PSS (checking, sorting and subsuming), finally compiled into the interview guide.

The interview guide includes a total of seven open-ended, narrative-generating main questions, divided into four thematic groups: I) entry, II) sitting interruptions in teaching, III) use of physically activating measures and teaching methods and IV) barriers to the implementation of physically activating measures and teaching methods (see Table 1).

**Table 1:** Questions of the interview guide used (examples of additional questions are in italics)

Introductory questions	If you look at your own working day, how do you alternate between sitting, standing, and walking?
	*Do you take extra care to interrupt your sedentary behaviour regularly?*
Sitting interruptions in teaching	What are typical reasons for students to stop sitting, stand, or move around in your courses?
Use of physically activating measures and teaching methods	When planning your courses, to what extent do you take care to interrupt the students’ sedentary behaviour?
	What measures, strategies, or teaching methods do you use in your courses to interrupt sitting or activate physical movement? *For example, do you make sure that discussions are held standing up?*
	*What other physically activating teaching methods do you know of?*
Barriers to the implementation of physically activating measures and teaching methods	What barriers and obstacles do you experience when implementing physically activating measures and teaching methods in courses?
	What barriers and obstacles do you expect for the implementation of physically activating measures and methods such as physical activity breaks, sit-stand desks or physically activating teaching methods in courses?

The interview guide was pilot tested in two pretest interviews with lecturers from the Heidelberg University of Education. One of these two interviews was conducted with a professor of educational science who is a proven expert in holistic learning processes and didactics. Immediately after this interview, the professor was asked about the quality and structure of the interview guide in the sense of an expert review. Only minimal adjustments were made to the interview guide after the pretest interviews, so both interviews were included in the main analysis. For example, we removed the following closing question from the final interview guide because it tended to irritate the lecturers and did not lead to any further insightful statements: “What is your general attitude towards sitting?”.

#### Data collection

The qualitative interviews were conducted from July 2017 to August 2017. Twelve interviews were conducted by the first author RR, PhD with experience in qualitative research and four by students (student assistants) from a BA Prevention and Health Promotion program under supervision. The interviews were held face-to-face at the lecturers’ workplaces. The interviews lasted approximately 15–25 min, were audio-recorded, and transcribed by the first author and one student assistant. The transcriptions were done verbatim, corrected for dialect and syntax, and the language was slightly smoothed [36]. The interviews were anonymized, so that no conclusions can be drawn about the personal data of the lecturers.

#### Data analysis

From May to October 2022, the first author (RR) analyzed the interviews, based on the structured content analysis according to Kuckartz [37], with the help of the software MAXQDA 2020 (VERBI GmbH, Berlin, Germany). In this type of analysis, categories are created, the interviews are analyzed with the help of these categories, the content is structured and summarized, and headings and subtopics are formed in the following way [37]:

1. initiating text work: marking important text passages and writing memos
2. development of main thematic categories along with the questions of the interview guide
3. coding of all available material with the main categories
4. compiling all text passages coded with the same main category
5. inductive determination of subcategories for further differentiation of the main categories
6. coding of the complete material with the differentiated category system
7. categorsy-based analysis and presentation of results

A deductive-inductive approach was chosen to derive the categories. The key questions of the interview guide provided us with the main thematic categories (deductive). These were further differentiated in the interview material based on subcategories and examples (inductive). The subcategories were formed with the help of thematically similar interview statements. These deductively and inductively derived categories formed the differentiated category system. The category system contains definitions, quotations, and coding rules to assign the text passages and to distinguish them from other categories. Data coding was mainly done by one of the authors (RR). Uncertainties in the analysis were discussed with the co-author (JB) until consensus was reached. The structured content analysis thus undertaken involved a reflective reading of the material and the familiarisation with the data led to a deeper understanding [37].

## Results

The sample (10 women and 6 men) consisted of twelve lecturers and four professors. All but one of the respondents worked in teacher training. The age structure of the sample is as follows: 20–29 (*n* = 1); 30–39 (*n* = 5); 40–49 (*n* = 4); 50–59 (*n* = 5); over 60 (*n* = 1). Due to the small size of the university (65 professorships, 168 lecturers) and the associated risk of identifying participants, no further personal data were collected or reported.

The identified themes out of the qualitative interviews were categorised into two main areas: Knowledge and use of physically activating measures and teaching methods and barriers to the implementation of physically active university teaching.

### Knowledge and application of physically activating measures and teaching methods

The analysis of the introductory question of the semi-structured interview shows that lecturers (still) pay little attention to physical activation or breaks from sitting when planning and carrying out their teaching. Many lecturers are simply not aware of a physically activating-sensitive perspective on their own teaching, as the following quote illustrates.

So, I represent the humanities (laughs) and in this respect, we haven’t actually realised that we should or ought to do something like this. (Int_4, paragraph 8).

It is therefore not surprising that just under half (7 out of 16) of the lecturers were initially unable to name any physically activating measures or teaching methods that they could spontaneously think of to their own teaching.

When looking at measures for physically active university teaching, the results show that only two of the sixteen lecturers stated in the course of the interview that they use physical activity breaks in their teaching to activate, mobilise, strengthen, stretch, or relax. Only one person utilises sit-stand desks in their teaching, which students can use to change their posture during the course.

Overall, it can be stated that more than two-thirds of the lecturers surveyed (11 out of 16 / 68.75%) know and use no or only a few (1–3) physically activating teaching methods (Int_1, Int_2, Int_3, Int_4, Int_5, Int_6, Int_7, Int_11, Int_13). Only two lecturers (Int_12, Int_15) have a broad(er) repertoire of physically activating teaching methods (5 or 7 methods). Table 2 systematically lists the identified physically active teaching methods that were derived from the interview data – that is, they were mentioned by the participants themselves. The method titles used in Table 2 were mostly provided by the lecturers interviewed. Where only a description of a method was given, we formulated a title for the method ourselves based on common designations and added it to the table. The descriptions of the methods in Table 2 are based on the statements of the lecturers, but were formulated in a condensed form by the authors of the study.

**Table 2:** Teaching methods used to reduce and interrupt sedentary behaviour in university teaching

Teaching methods for interrupting sedentary behaviour	Description	Number of references
Group work	Physical movement results from coming together in a (new) working group	12
Change of learning location	Students leave the classroom and temporarily use the stairwell, corridor, courtyard, and neighbouring green spaces as a place of learning	4
Presenting in front of a seminar group	Individual students stand in front of the learning group and present the results of the group work or give presentations	4
Positioning in the room / line-up	Students position themselves on a scale on the floor or stand in front of a specific wall poster in the room and thus express their agreement/disagreement, interest in a topic/task, (dis)satisfaction with the learning process …	4
Sketches on blackboards	Individual students come to the board to present solutions or brainstorm ideas	3
Fishbowl method	Students organise themselves into an inner and an outer circle to lead the discussion. Those who wish to actively participate in the discussion move to the inner circle	2
Learning circuit	Work assignments of various kinds are laid out at different positions in the room, which the students work on one after the other	2
Gallery-Walk / Vernissage	Working groups display their results in the classroom (e.g. on a flip chart). All students walk around the room and look at the results of the other groups	2
Learning counter	Learning materials are laid out on a "counter". Students go to the counter and select the materials they want to work on from those available	2
Get worksheets yourself	Worksheets are not handed out but must be picked up by the students themselves	1
Extramural places of learning / excursions	The learning group leaves the classroom for a " teaching tour" to learn "on-site"—e.g. at a historically significant location	1
4-Corner-Method	Different points of view on a topic are distributed in each corner of the room. The students assign themselves to the statement that they are most likely to agree with by moving to the corresponding corner of the room	1
Jigsaw method	Learning content to be taught is divided into different groups, which first acquire expert knowledge for their part. Students from the various expert groups then come together (again) in puzzle groups to teach each other their content	1
Double circle	Students stand facing each other in an inner and an outer circle and talk to each other about a topic. After a short time, the circles move in opposite directions. Another exchange phase takes place in a new constellation of students	1
Pair interview	Students walk around the classroom in pairs and discuss a given question. After a limited time, they can change partners	1
Role play	A topic is presented in a playful way—using the body	1
Experiments	Movement occurs when setting up and personally carrying out experiments on scientific subjects—usually standing at laboratory tables	1
Use room walls as work and presentation areas	Work assignments, solutions, text excerpts, definitions, and work results … are placed/processed on the walls in the form of slips of paper or flip charts. Students must stand up and walk over to get information or work on tasks	1

The lecturers mainly mentioned teaching methods that incidentally lead to interruptions in sitting during their courses, without being consciously intended. In this context, common teaching methods were identified: moving during group work (12 references), giving a presentation or speech while standing in front of the learning group (4 references), and writing on the blackboard (3 references). All of these methods align with physically activating teaching approaches.

However, the lecturers themselves note that these three typical but unintentional interruptions to sitting during the course either only occur rarely, only lead to physical activity for individual students — and not for the whole seminar group (e. g. during presentations and writing on the blackboard) or the physical activity they generate is very low.

So, the minimum amount of physical activity during group work is that the students stay in their seats and just turn their chairs. (Int_15, paragraph 12).

Thus, the central physically activating teaching method in university courses (group work) often turns out to be just another reason for sitting, which hardly contributes to the interruption of sitting / physical activation. The situation is different when group work is combined with a change of learning location. Four of the lecturers surveyed stated that they occasionally allow students to leave the classroom during group work and temporarily use the stairwell, corridor, courtyard, or neighbouring green spaces as a place to study. The combination of group work and a change of learning location then has a significantly higher potential for physical activity.

### Barriers to the implementation of physically activating teaching methods and measures in university teaching

Five sub-themes were identified as barriers to the implementation of physically active teaching. They can be summarised as follows:

- Uncertainty in implementation: Lack of methodological knowledge / Lack of pedagogical- didactic concept
- Lack of space: too small rooms / too many students
- Lack of time: high volume of learning content / precious learning time
- Students’ unwillingness to move: Physical laziness or pronounced sitting habit?!
- Organisational social norms: Teaching culture based on sitting / Student expectations

### Uncertainty in the implementation of physically activating teaching methods and measures

Most of the university lecturers interviewed have completed a teacher training program. Despite their profound pedagogical-didactic and methodological knowledge, many lecturers stated that, in addition to a lack of knowledge of physically activating methods and measures, they also had uncertainties regarding the pedagogical-didactic and methodological implementation of these in university teaching.

So, I would simply think that my main focus is on teaching the students some didactic content. Partly through lectures, partly by having them deal with tasks themselves and I would find it difficult at first glance because I also have little experience in thinking about how I can now implement this and somehow integrate walking or standing at the same time. (…) In other words, the barriers, to come back to your question, are mainly that I lack a concept of how I can integrate this in a meaningful way without having to accept a loss of content. (Int_10, paragraphs 16–18).

In addition to this general didactic and methodological uncertainty regarding the integration and implementation of physically activating teaching methods and measures, many lecturers emphasised that they consider physical learning approaches to be particularly suitable for younger pupils in schools and that they are not really an appropriate option for adult learners in university teaching.

So, in everyday school life, we often did physical activity with ritualised songs that addressed all areas of the body, especially the shoulders and the similar. I’m not quite sure to what extent you can make that usable for university students without it becoming silly (laughs), you certainly need other methods. (Int_11, paragraph 20).

The uncertainty about implementation, but also the lecturers’ further interest in this topic, was often expressed in the explicit desire for concrete methodological and pedagogical-didactic guidance or further professional development.

### Lack of space: mismatch between (too small) room size and (too) large number of participants

Lecturers noted that the physical classroom environment, particularly the lack of space for physically activating activities, made it difficult for them to integrate physically activating methods and measures into their courses. Lecturers reported that this problem was most evident in larger rooms with fixed rows of seats.

However, lecturers also relativised the scope of the specific lecture hall problem as a fundamental obstacle to dynamic university teaching.

"Of course, there are spaces and events where physical activity is not so convenient. Lectures in the lecture hall with rows, that is indeed awkward. But I believe that only affects a small percentage of the teaching one encounters." (Int_12, paragraph 20).

According to lecturers, the frequent disproportion between the small size of the teaching room and the high number of students attending courses also hinders physical activity. In particular, the lecturers interviewed, who held large courses with a high number of students, emphasised the disproportion between the number of students and the size of the room as a key obstacle to the implementation of physically activating teaching methods and measures in courses.

I think one of the main problems is that the rooms are extremely crowded. We often have the problem that not all students even have a table and chair. And if they do manage to squeeze a chair into a corner somewhere, there’s not much space to move around. So maybe the ones who have found a place on the floor are the ones who are most likely to move (laughs). (Int_11, paragraph 16).

Lecturers of small seminars were much more optimistic in their assessment of the implementation of physically active teaching due to the generally significantly lower number of participants in courses.

### Lack of time

The effect of physical activity in reducing learning time was seen as a further barrier to its implementation in teaching. Against the backdrop of a high density of teaching content, the lecturers reported their concern that they would no longer be able to teach the subject matter in the scheduled time if learning time was lost due to physical activity. For the lecturers, this concern about loss of time due to physical activation related primarily to the classic 90-min course format. For double-hour courses or block courses, on the other hand, they saw much more favourable opportunities to integrate physical activity measures and teaching methods into their course concept in terms of time.

And what I haven’t done yet is a block course. I think that could be designed much better or somehow incorporate physically activating methods, because of course, you have to keep changing the teaching format when you sit together for eight hours. And I think there’s more opportunity to do that than in 90 min, which, depending on what you want to do thematically, is not as long as it might sound now. (Int_1, paragraph 22).

The lecturers also made a connection with the problem of "lack of space", which can delay the timing of physically activating teaching methods and measures and thus exacerbate the time problem:

If I’m in a compulsory course where all our student teachers have to sit through once, then the seminar is 40 to 45 people in a room designed for 30 to 35 people. Then of course it’s difficult if the students don’t all have a seat and have to sit in the second row at the back. Then you also save yourself a bit of movement, because then you know, OK, now it’s full of confusion until someone is in another position, which means that the 90 min are wasted again. (Int_1, paragraph 24).

However, not all the lecturers shared the previously outlined view of physically activating measures and teaching methods as reducing learning time. Two lecturers explicitly stated that they did not recognise a time problem in the implementation of physically activity in teaching:

Otherwise, I wouldn’t see so many barriers, maybe even none at all. Of course, you can always say that it takes time away from me because the students have to find each other first and then it’s more restless or something. On the other hand, it’s also an illusion to believe that they’re always attentive just because they’re sitting down (laughs). (Int_12, paragraph 20).

### Students’ unwillingness to move

Five of the sixteen lecturers interviewed reported that they had already experienced that students were sometimes reluctant to engage in such physical activations when setting physically activating impulses as part of their courses. The lecturers perceived this as an obstacle to the implementation of physically activating methods and measures, which they said had also prevented them from implementing further impulses of physical activity in the past.

This reluctance to move on the part of students is observed by lecturers not only when using (large) methods of physical activation with larger impulses of physical activity (e.g. excursions), but also with small activations, such as the frequently used group work (Int_16, paragraph 16).

I've noticed that when I say: "Now please form groups" and I tell four students to sit together, all four of them start to say: "Oh, the others should come here. So, they really don’t want to move. And, basically, it's already happened to me, in a huge room, I’ve had two or three groups that were very close together and who kept disturbing each other during group work and I then said: "Yes, but the room is so big, you can get up and go there." And then they’d say: "Oh, no, no, we’d rather stay here" or something like that. I’ve already observed that. (Int_7, paragraph 36).

Some lecturers try to counter this reluctance to move with methodological variation:

I think there is always the moment when you want to get a group that is sitting down moving, the hurdle is great. That always has to be done methodically and you also notice, or I notice when I say: "Mix and mingle", then the students stay where they are. They just turn their chair round. The least I sometimes say is: "Now go with someone you haven’t spoken to yet this semester." Then I don’t know how they do it, but at least a few more students move around. (Int_15, paragraph 22).

Other lecturers see the barrier less in a fundamental unwillingness to be physically active on the part of the students, but rather in a pronounced habit of sitting during courses, which becomes more habituated over the course of the program.

Whether a general reluctance to be physically active or have an acquired habit of sitting, the role of a physical animator that falls to them represents a significant obstacle to realisation.

Sometimes, I have to say, the reaction of the students really put me off: "Oh, now we have to do it again". This prescriptiveness is a barrier for me. (Int_13, paragraph 26).

### Organisational social norms

The perception of what was considered "normal" practice and behaviour in university teaching influenced the willingness and feasibility of teaching with physical activation. Half (*n* = 8) of the lecturers reported a teaching culture that did not support regular stimuli of physical activity. They noted that concerns about being seen as "weird" or feeling uncomfortable prevented them from integrating physically activating teaching methods and measures more strongly into their courses, as sitting was simply the norm in university teaching.

If it were common practice [to integrate physical activity impulses into teaching; author’s note], then it wouldn’t be so difficult to initiate. If everyone did it and the students were used to it and found it normal, then there would no longer be a barrier. (Int_8, paragraph 40).

It was also reported that such physical activity-impeding normative concepts were already biographically characterised in lecturers during their own studies and that these still had an effect in their current role as lecturers.

You just sit in a seminar or lecture. I couldn’t have imagined simply standing up in a lecture as a student. I have to say that even as a lecturer, it would be strange for someone to stand up all of a sudden. Why would he do that? (laughing) I wouldn’t do that myself. (Int_3, paragraph 48).

Lecturers also reported several times that they were worried about making a fool of themselves in front of the students by using physically activating impulses in teaching, or that they would need explanation because this could violate the established sitting expectations of the students.

How you can somehow integrate physical activity in a meaningful way so that it would actually be perceived as a variety and that I’m not somehow in need of explanation to the students, because that might actually be the second barrier. So, A, I don’t really have an idea of how I can implement it, but B, I would also think that the students would perceive it more as actionism. I don't know if that’s actually the case, but I could imagine it. (Int_10, paragraphs 19–20).

In this context, one lecturer suggested carrying out physically activating methods and measures primarily in the lower semesters, as students’ expectations of sitting are not yet so pronounced here.

## Discussion

To the best of our knowledge, this qualitative study is the first to give insights into the subjective perspectives of lecturers on physical activation and interruption of sedentary behaviour in university teaching. It examines the questions of whether university lecturers know and use physically activating teaching methods and measures and which barriers they generally experience or expect when implementing such methods and measures. The results show that the lecturers hardly know and use any physically activating teaching methods and measures. However, with the implementation of group work, only one "cause for physical activity" was identified which is regularly and widely used in university teaching. Conventional measures such as physical activity breaks and sit-stand desks, on the other hand, were hardly used. Nevertheless, the 18 identified teaching methods results in a variety of potential methodological options and might be used to pursue an approach for physically active university teaching that is primarily driven by pedagogical-didactic consideration. The main barriers to the implementation of physically activating teaching methods and measures were identified as lecturers’ uncertainty when using such methods and measures and the limited physical space available. In addition, organisational and social norms, students’ unwillingness to be active, and a lack of time were further identified as barriers.

Our study shows that lecturers did not use conventional measures to interrupt sitting and teach in a physically activating way. Only two lecturers state that they use physical activity breaks in their courses. This low level of implementation confirms a study result according to which the implementation of physical activity breaks in teaching is hardly widespread at German universities [38]. According to this study, physical activity breaks are only practised in a more widespread manner at 18 out of a total of 204 universities and universities of applied sciences in Germany. One reason for their low practical application could be traced back to the barrier of “lack of time”. While the use of sit-stand desks and the application of physically activating teaching methods are characterised by time-saving physical activation, physical activity breaks are invariably associated with a loss of teaching time by their very nature. The measure of physical activity breaks thus violates a central core concern of lecturers to be able to effectively convey a lot of teaching content in the time given. We will discuss this pedagogical perspective below in more detail.

The use of physically activating furniture in this study (only one mention) is disillusioning and can be attributed to the fact that at the time the interviews were conducted, there was only one movement-friendly seminar room at the university studied, which was partially equipped with sit-stand desks. Only one of the lecturers surveyed was teaching in this study room, which was solitary available to sports lecturers, and therefore had access to this measure at all. It was after the study that another movement-friendly teaching room had been completed, which could also be used by lecturers from other subjects. This was introduced at the university by great financial expense — and with financial support from the Federal State of Baden-Württemberg (quality assurance funds): the stand-up teaching lab [6, 39]. This represents a further illustration of the cost-intensive nature of this measure and the associated difficulty of scaling it up.

In direct comparison with the conventional measures of physical activity breaks and sit-stand desks, physical activating teaching methods appear to be advantageous because they are cost-effective, close to the subject and save learning time. Therefore, physical activating methods seem like a promising practice for a pedagogical-didactic approach to physically active university teaching – in addition to physical activity or standing breaks and the use of physically activating furniture (e. g. sit-stand desks).

In sum, the lecturers surveyed identified 18 physically activating teaching methods for interrupting sitting. Despite the high total number of physically activating teaching methods identified, it should be noted that this approach is also still rarely taken by individual lecturers. Group work is the only method that leads to interruptions in sitting that is implemented to a certain extent and frequency in university teaching. However, lecturers also state that group work often does not lead to real interruptions to sitting or physical activity, but merely to "moving chairs". The majority of the other identified physically activating teaching methods were only mentioned by one to a maximum of two lecturers. Our results give a rationale to the prevalence studies that emphasise the strong sedentary orientation and implementation of university teaching while failing to take a closer look at the reasons for possible interruptions to sitting [1, 3, 40].

The compilation of physically activating teaching methods identified here can serve as an initial orientation. They synchronise physical activity and learning in university teaching in terms of time — for example, when students walk through the classroom in pairs during a pair interview and exchange views on a given task. The time with physical activity is also learning time and serves an overarching pedagogical-didactic goal. Physically activating teaching methods are thus characterised by the fact that they lead to a double activation of the students — physically as well as cognitively — while pursuing specific learning objectives [6, 24]. With this approach to interrupt sedentary behaviour in a university context we take a pedagogical-didactic perspective as the primary goal by incidentally promoting health. This idea is linked to stealth health considerations [24, 41] as the motivation to implement a health-orientated strategy into the overarching aim of designing learning-efficient university teaching.

The other research question in our study was about the barriers that lecturers generally experience or expect when implementing physically activating teaching methods and measures in their teaching practice. Despite our study revealed an openness to physically activating teaching methods and measures, there is at the same time a lack of knowledge, familiarization and expertise regarding the purpose and concrete implementation options, which leads to uncertainty and a lack of sensitivity among lecturers when it comes to integrating physical activity into the teaching of lectures. These uncertainties combined with large amount of content to be taught relate to fundamental pedagogical-didactic and methodological questions about the right time, the right dosage, and, above all, the goal-congruent combination of physical activation with the prioritised teaching of subject content within the framework of their courses. The ability to teach with physical activity is therefore a complex skill that lecturers still need to acquire. This is a significant barrier to the widespread implementation of physically active university teaching. However, it is consistent with the findings from the school sector that teachers are unsure about implementing health didactic principles and must first acquire the relevant skills through appropriate further professional development [42].

Our study highlights that lecturers are the crucial bottleneck through which the innovation of pedagogically and didactically orientated physically active teaching must pass. For this to succeed, it is necessary to strengthen the knowledge, sensitivity, and acceptance of lecturers for physically activating methods and measures and at the same time to enable them to realise these competently in their teaching. However, this complex ability cannot be built up by reading a book alone but also requires participation in further training in higher education didactics, which has been proven to increase the teaching competence of lecturers comprehensively and broadly in the areas of knowledge content, skills, attitudes, and behavior [43]. Taken a stealth health approach with a strong pedagogical-didactic emphasis to improve university teaching with physically activating methods is innovative as well as promising and should be examined in further studies with lecturers and students.

The lack of space in classrooms has proven to be another significant barrier. The current classroom sizes and facilities hardly allow for any physical activity, particularly due to the tables and chairs used. They are primarily designed for sitting still in lecturer-centred lessons and hardly support the implementation of physically-activating methods and measures due to the lack of space for movement, which is also confirmed by studies from the school context [33, 34]. This obstacle to physical activity seems to be the easiest to overcome for small seminar groups. For large courses with many students, the lack of space for physical activity severely restricts the possibilities for implementing methods and measures that encourage physical activity — especially if they take place in lecture halls with fixed sitting in narrow rows of seats.

Our study also shows that the sitting posture at universities is a strongly normative behaviour that is not questioned by lecturers and is seen as the correct and culturally established way to deliver university teaching. This high importance of the norm of sitting is also found in other areas of society such as school or office work [26, 44]. It is also interesting to note that the university sitting norm in courses is not only fed and consolidated by the individual ideas and expectations of the lecturers, but also by so-called "expectation expectations" [45]. These are expectations that relate to the expectations of a counterpart — in our case the (sitting-related) expectations of the students — and that contribute to the stabilisation of social situations (in this case courses). Against the background of this strong organisational social norm, it seems appropriate to also implement organisational countermeasures and guidelines in the sense of a behavioural and contextual approach [46]. In the workplace context, measures range from individual awareness raising to the supportive attitude of managers [47, 48]. Applied to the university context, this means not only conducting university-wide awareness campaigns regarding the risks of sedentary behaviour and the benefits of physically active university teaching for students and lecturers but also support from the rectorate to enable changes to teaching spaces or the purchase of furniture that promotes physical activity. Also, in this case it might be useful to take a stealth health perspective in account to highlight the improvement of the pedagogical-didactic quality of teaching instead of taking an isolated health perspective.

It should also be noted that the lecturers had the perception that students were reluctant to physically activity during courses. Further research is needed to clarify whether this is a general reluctance to physical activity or rather an established habit of sitting in teaching situations throughout their entire educational biography. Furthermore, it would be interesting to learn more about the subjective views of university students regarding more physical activity in teaching and whether this is in accordance with the subjective views of lecturers. However, the design of teaching, which is massively geared towards sitting, probably support this situation, as does the affordance of the social (norm to sit in courses) and physical environment (room furnishings), which invite students to adopt sedentary behaviour [25]. At this point, the interaction of the five identified barriers becomes clear. The barriers not only act in isolation, but also in their mutual interplay, touching on individual, social, environmental, and organisational levels. Therefore the findings from this study support the usefulness of conceptual and multidimensional models like the Heidelberg Model of Physically Active University Teaching [6]. This model can serve as an interventional starting point to overcome identified barriers (see Fig. 1). A closer examination of the model reveals that all barriers align with its five components as outlined in the introduction. In this conceptual framework, professional development for lecturers appears as a key component for successful implementation. It addresses not only the uncertainties regarding implementation but also the lack of methodological and didactic knowledge, the social norms associated with sitting, and the lack of time – four of the five barriers identified. Physical activity-friendly classrooms represent an approach for solving the barrier of lack of space, whereas physical activity breaks and physically activating teaching methods address the barrier lack of methodological and didactical knowledge and, in particular, learning methods also address the barrier lack of time. Finally, curriculum-based study programmes for physical activation are tailored to the barriers of students’ unwillingness for physical activity and social norms based on sitting.

### Strengths & limitations

One of the strengths of this study at the sampling level is the breadth of subject areas included. Lecturers in math, physics, languages, education, history, sport, and sociology were interviewed. This means that the natural sciences, humanities, and social sciences were considered. Another strength is the variety of backgrounds of lecturers, which covers all relevant levels (professors, academic staff) and thus contributes to the generalisability of our findings. The genders were also evenly represented and there was a broad age spectrum.

The interpretation of our findings has also taken several limitations into account. First, this study interviewed only lecturers from one university. The transferability of the results to all higher education institutions and universities is limited because the considered university comes from a pedagogical context. This suggests possible selection effects that mask the actual awareness and feasibility of physically activating measures and methods in university teaching. It can only be hypothesized that this problem is even more pronounced at other (non-pedagogical) universities. In addition, we have also translated quotes from German into English. However, the translation does not detract from the authenticity of the statements. Moreover, the coding was conducted by the first author, while trained students supported the interview process. A second coder was not applied, as the extracted data —teaching methods and barriers to implementation — left little room for interpretation. The clearly distinct categories, such as lack of time, lack of space, or organizational norms, allowed for a reliable assignment. In cases of uncertainty (*n* = 3), coding decisions were discussed and resolved between the first and last author. However, we acknowledge that the use of multiple coders is a common quality criterion in qualitative research and recognize this as a limitation of our study. Furthermore, the influence of the researchers’ characteristics like research experience, teaching experience, age, and gender on the interpretation of the interviews should be considered. Thus, for the two authors involved in the data interpretation, sedentary behaviour is not only one of their central research topics, but they have also been implementing physically activating measures and methods in their own teaching with great passion and conviction for many years. However, the aim of the qualitative data analyses was relatively clear to define by identifying methods and reasons for implementing active teaching procedures in lectures. In addition, it should be noted that our study reflects the situation in 2017, which relates to purely on-campus teaching. In recent years, the COVID-19 pandemic has given a massive boost to remote teaching and the digitization of university teaching. At the same time, it is highly likely that lecturers are even less familiar with physically activating teaching methods for online-based teaching than compared to the face-to-face context.

## Conclusions

This study opens up new perspectives for interventions to reduce sitting time and promote physical activity in university teaching with a stealth health perspective. It was based on a primarily pedagogical-didactic approach to assure a "high-quality education" as the core concern of higher education teaching. In this context, we have identified measures, methods and barriers to interrupting sitting time. Whether these physically activating measures and methods can actually be used and which conditions would be needed for this should be further investigated. In addition to the initial findings on physical activity breaks and physically activating furniture, the physically activating teaching methods in particular need to be examined more closely to see whether they can be implemented and whether they are actually accepted by students. Intervention- and evaluation concepts should be developed for this purpose. Initial conceptual considerations for this can be found in the Heidelberg model of physically active university teaching [6].

## Data Availability

The data described in this article are available from the corresponding author on reasonable request.
